# The enigmatic role of tumor dormancy cells in gynecologic cancers

**DOI:** 10.3389/fimmu.2026.1836770

**Published:** 2026-05-15

**Authors:** Aizhen Fu, Zhen Ma, Kai Zou, Feiyuan Wu, Xinxin Zou

**Affiliations:** 1 The Second Affiliated Hospital of Guangdong Medical University, Zhanjiang, Guangdong, China; 2 The Affiliated Hospital of Guangdong Medical University, Department of Gynecology, Zhanjiang, Guangdong, China; 3 Department of Gynecology, Central People’s Hospital of Zhanjiang, Zhanjiang, Guangdong, China; 4 Hubei University of Medicine, The Fifth Clinical Medical College, Shiyan, Hubei, China

**Keywords:** gynecologic tumors, immune evasion, tumor dormancy cells, tumor drug resistance, tumor recurrence

## Abstract

Gynecologic cancers (GCs), including ovarian, cervical, and endometrial cancers, represent a substantial global health burden, characterized by high rates of recurrence, therapeutic resistance, and metastatic dissemination. Tumor dormancy—a state in which disseminated tumor cells (DTCs) persist in a non−proliferative, quiescent phase, thereby evading conventional therapies and immune surveillance—constitutes a critical yet often underestimated driver of these clinical challenges. This comprehensive review systematically integrates the multifaceted roles and current research landscape of dormant tumor cells in gynecologic malignancies. The core innovation lies in a three−level analytical framework that examines dormancy through intrinsic molecular switches, extrinsic microenvironmental remodeling, and cross−cancer type comparisons. Specifically, the mechanisms governing dormancy initiation, maintenance, and reactivation are delineated for cervical, ovarian, and endometrial cancers. Several key conclusions emerge from this synthesis. Common regulatory hubs across gynecologic cancers include hypoxic conditions, cell−cycle regulators such as the DREAM complex, stemness−associated pathways exemplified by the HIF−1α/PLD2 axis, and stromal cell interactions, notably cancer−associated fibroblast−extracellular matrix crosstalk. Dormant cells further orchestrate sophisticated immune evasion strategies, including downregulation of major histocompatibility complex class I and upregulation of immune checkpoint molecules, thereby establishing a reservoir of drug−tolerant persister cells that drive post−treatment relapse and acquired resistance. Notably, substantial heterogeneity exists across different gynecologic cancer types: ovarian cancer engages the most diverse repertoire of dormancy−related pathways, while uterine sarcoma remains a conspicuous research gap. Collectively, this review establishes the dormant tumor cell reservoir as a promising therapeutic target to prevent recurrence and overcome therapy resistance. Specific actionable targets—including Dyrk1A, PLD2, LATS1/2, and Egfl6—are proposed, providing a theoretical foundation for the development of novel diagnostic tools and therapeutic strategies aimed at improving long−term outcomes for patients with gynecologic malignancies.

## Highlights

- Tumor dormancy cells represent a critical biological basis for treatment failure and recurrence in gynecologic cancers. These cells enter a quiescent state, evading conventional radiotherapy, chemotherapy, and immune surveillance, and can remain latent for years before reactivating to cause disease relapse.
- In gynecologic malignancies such as cervical, ovarian, and endometrial cancers, dormant cells drive radioresistance, chemoresistance, and immune escape through distinct pathways, posing significant clinical challenges.
- Current research focuses on developing novel strategies to target these cells, including direct elimination, induction of permanent dormancy, or reactivation followed by conventional therapy, supported by advanced technologies like organoids and single-cell sequencing to accelerate clinical translation.

## 1 Introduction

Gynecologic cancers (GCs) encompass a diverse group of malignancies affecting the female reproductive system, including ovarian, cervical, and endometrial cancers, which collectively pose significant health threats with increasing incidence rates and high mortality due to late diagnosis, aggressive progression, and the development of therapeutic resistance (1). Despite advancements in surgical techniques, chemotherapy, and radiotherapy, a substantial proportion of patients experience disease recurrence, often years after initial successful treatment, leading to a dismal prognosis (2). This persistent clinical challenge is increasingly attributed to the phenomenon of tumor dormancy, a complex biological state where cancer cells, either at primary or metastatic sites, enter a quiescent, non-proliferative phase, effectively evading detection and the cytotoxic effects of conventional therapies that primarily target rapidly dividing cells (3–5).

Tumor dormancy is broadly categorized into cellular dormancy, where individual disseminated tumor cells (DTCs) remain quiescent, and tumor mass dormancy, characterized by a balance between proliferation and apoptosis within a microscopic lesion (6). Understanding the intricate mechanisms governing the entry, maintenance, and eventual reawakening of these dormant cells is paramount for developing effective strategies to prevent disease relapse and overcome therapeutic resistance in GCs. This paper aims to provide a systematic and comprehensive review of the current research status and the pivotal role of tumor dormancy cells in gynecologic tumors. We will explore the interplay between intrinsic cellular programs and the dynamic tumor microenvironment (TME) in regulating dormancy, specifically examining its implications for tumor recurrence, drug resistance, and immune evasion within the contexts of cervical cancer, ovarian cancer, endometrial cancer, and, where applicable, uterine sarcoma. By integrating insights from recent literature, we seek to highlight critical research gaps and propose future directions for targeting dormant cell populations to improve clinical outcomes for patients with gynecologic malignancies.

## 2 Tumor dormancy: mechanisms and microenvironmental influences

Tumor dormancy represents a dynamic and multifaceted biological state crucial for cancer progression and recurrence. It is characterized by the persistence of viable, non-proliferating cancer cells that can remain undetectable for extended periods before reactivating to form overt metastases (3, 6, 7). The regulation of dormancy involves a complex interplay of intrinsic cellular mechanisms and extrinsic cues from the tumor microenvironment (TME) (8, 9).

### 2.1 Intrinsic cellular mechanisms of dormancy

At the cellular level, dormancy is primarily driven by cell cycle arrest in the G0/G1 phase, a state that confers resistance to proliferation-targeting therapies (10, 11). This quiescent state is orchestrated by a network of gene regulatory pathways. Reduced levels of the urokinase receptor (uPAR) in human carcinoma cells induce G0/G1 arrest and dormancy through decreased adhesion to fibronectin and reduced ERK1/2 activation, indicating that insufficient uPA/uPAR/α5β1 complexes prevent sustained tumor growth (12). The F-box protein Fbxw7 maintains quiescence of dormant breast cancer cells; its ablation disrupts dormancy, promoting proliferation and enhancing chemosensitivity, which identifies Fbxw7 as a potential therapeutic target to prevent recurrence (13). Conversely, low levels of Sox2 are required for melanoma tumor-repopulating cell dormancy, and Sox2 depletion triggers dormancy exit and apoptosis via p53-caspase3 activation (14). RGS2 mediates translational control by inducing eIF2α phosphorylation, promoting dormancy-like phenotypes and high survival capacity in slow-cycling cancer cells (15). The nuclear receptor NR2F1, a key dormancy-associated transcription factor, reduces therapeutic efficacy and suppresses tumor proliferation while sustaining mTORC1 transcriptional regulation, highlighting its role in therapeutic resistance (16). NRF2 activation, often linked to oxidative stress, promotes recurrence of dormant tumor cells by inducing metabolic reprogramming for redox homeostasis and nucleotide synthesis (17). MacroH2A variants, especially macroH2A2, enforce a discrete dormancy program in disseminated cancer cells by inhibiting cell cycle and oncogenic signaling while upregulating dormancy- and senescence-associated cytokines, thereby impeding metastatic growth (18). Epigenetic modifications, such as histone H3.3 deposition, control dormancy entry and exit in disseminated cancer cells; H3.3 incorporation represses SKP2, leading to p27 accumulation and cell cycle arrest (10). Collectively, these genetic and epigenetic changes, together with metabolic adaptations such as mitophagy that protect cancer cells from chemotherapy during dormancy, underscore the complex intrinsic regulation of this state (3, 19).

Collectively, while multiple intrinsic regulators have been identified, a core triad emerges as dominant gatekeepers of cellular dormancy in gynecologic cancers: DREAM complex-mediated cell cycle arrest、NR2F1-driven transcriptional reprogramming and NRF2-mediated redox homeostasis. These three nodes converge to enforce a reversible G0/G1 arrest and confer broad therapy resistance. Other factors (e.g., Fbxw7, Sox2, RGS2) appear to play more context-dependent roles in specific tumor types or stress conditions.

### 2.2 Extrinsic microenvironmental influences on dormancy

The tumor microenvironment (TME) is a critical determinant of dormancy, influencing its initiation, maintenance, and reactivation (8, 9, 20). Interactions between dormant cells and the extracellular matrix (ECM) are particularly important. For instance, fibronectin, organized by tumor cells, can maintain a dormant breast cancer population, with its degradation by MMP-2 being necessary for cancer cell outgrowth post-dormancy (21). Similarly, the ECM of targeted organs can either maintain dormancy or reactivate growth, emphasizing its crucial role in late metastasis (22). Lung inflammation, often caused by factors like tobacco smoke, can awaken dormant cancer cells by inducing neutrophil extracellular traps (NETs), whose proteases cleave laminin, triggering dormant cell proliferation (23). Hypoxia, a common feature of the TME, is a potent inducer of dormancy, leading to tumor cells that are resistant to chemotherapy (20). In HPV-positive cancers, hypoxic conditions induce a reversible dormant state with downregulated E6/E7 expression, protecting cells against chemotherapy and virus-specific therapies (11). The bone microenvironment, for example, can drive dormancy escape through osteoclast-mediated bone resorption, which releases growth factors, and the metabolic support provided by bone marrow adipocytes, while osteoblasts can also maintain cancer cell dormancy through secreted factors and direct cell-to-cell contacts (24, 25).

Immune cells within the TME also significantly influence dormancy. For instance, host immunity, particularly an IFN-γ-rich microenvironment, can control tumor dormancy by enhancing KLF4-mediated SLURP1 production in malignant cells, while NK cell immune surveillance can be inactivated through the CD200-CD200R1 mechanism (26). The DREAM repressor complex, dependent on Dyrk1A, has been implicated in cancer cell dormancy, with its inhibition leading to increased DNA synthesis and cell death, suggesting Dyrk1A as a therapeutic target (27). The concept of senescence, a growth arrest state, is closely intertwined with dormancy. While chemotherapy and radiotherapy often induce senescence, tumor cells can recover from “pseudo-senescence,” allowing them to evade therapy, survive in a dormant state, and contribute to recurrence (28–30). This state can be influenced by soluble factors like IGF1, where a synthetic mimetic of heparan sulfate hexasaccharide, G2.2, has been shown to induce dormancy in wild-type IGF1R cells and inhibit tumor recurrence in xenografts, potentially through IGF1R inhibition (31). The dynamic interplay between these intrinsic and extrinsic factors highlights the complexity of tumor dormancy and the need for comprehensive approaches to understand and target it. Among the diverse microenvironmental signals, hypoxia and ECM stiffness emerge as primary inducers of dormancy entry, while inflammation (e.g., NETs) and niche-specific growth factor gradients (e.g., from bone or omentum) serve as dominant reactivation triggers. Immune surveillance, particularly the CD200-CD200R1 and IDO-Kyn-AhR axes, acts as a continuous rheostat that maintains the dormant state until disrupted by pro-inflammatory cues.

### 2.3 Advanced technologies for studying dormancy

The intricate nature of tumor dormancy necessitates advanced research methodologies. Single-cell and long-read sequencing, combined with sophisticated analysis methodologies and artificial intelligence, are being employed to provide a comprehensive understanding of cancer dormancy, allowing for the identification of quiescent cells and their unique molecular profiles (5, 32, 33). Organoid technology offers *in vitro* models that closely replicate tumor cell architecture and heterogeneity, preserving histological and molecular characteristics of primary tumors, which is invaluable for studying dormancy mechanisms, biomarker discovery, and personalized therapeutic strategies in gynecologic cancers (1, 34). Furthermore, bioprinting is being utilized to create tunable 3D co-culture systems that mimic the tumor microenvironment, enabling the identification of soluble factors and pathways regulating dormancy and reactivation, facilitating high-throughput screening of therapeutics to prevent late recurrence (35). These technological advancements are crucial for unraveling the mysteries of dormancy and translating findings into clinical applications.

## 3 Tumor dormancy in gynecologic cancers: a specific focus

Gynecologic cancers (GCs) present unique challenges due to their heterogeneity, aggressive nature, and propensity for recurrence and metastasis. Tumor dormancy plays a critical, yet often underappreciated, role in the clinical trajectory of these malignancies, influencing treatment outcomes and patient survival (1, 36).

### 3.1 Cervical cancer

Cervical cancer (CC), largely driven by human papillomavirus (HPV) infection, can exhibit dormancy, particularly under hypoxic conditions. Hypoxia in HPV-positive cancer cells induces a reversible dormant state characterized by downregulated E6/E7 oncogenes and a lack of senescence, which protects these cells from chemotherapy and virus-specific therapies. This impaired mTOR signaling, mediated by the REDD1/TSC2 axis, prevents senescence and enhances therapeutic resistance, suggesting that hypoxic HPV-positive cells can serve as reservoirs for recurrence upon reoxygenation (11). Beyond hypoxia, specific molecular pathways contribute to dormancy-mediated radio resistance in CC. Apoptotic tumor cell-derived extracellular vesicles (apo EVs) containing MTA1 have been shown to confer radio resistance by activating the p-STAT1/NR2F1 signaling axis, thereby promoting cellular dormancy. This transfer of MTA1 via caspase-3 activation and apo EVs leads to increased radio resistance, indicating that targeting MTA1 or inhibiting p-STAT1 could enhance radiosensitivity and offer a novel therapeutic strategy for CC patients (37). Furthermore, necroptosis, a form of programmed cell death, has a dual role in CC, promoting tumor aggression by enhancing VEGFA expression via the JAK2-STAT3 pathway, but also modulating the immune microenvironment by activating T-cell pathways and promoting Jurkat T cell infiltration. While bevacizumab can induce necroptosis, resistance may occur, suggesting that combining bevacizumab with necroptosis inhibitors could reduce VEGFA and improve outcomes (38). The TME also plays a role, with neutrophil extracellular traps (NETs) influencing CC progression by attracting tumor cells, stimulating further NET release, and altering tumor cell behavior, increasing invasiveness and metastasis through immune modulation and epithelial-mesenchymal transition (39).

### 3.2 Ovarian cancer

Ovarian cancer (OC), particularly high-grade serous ovarian carcinoma (HGSOC), is the most lethal gynecologic malignancy, largely due to late diagnosis, extensive peritoneal metastasis, and the rapid development of chemoresistance and recurrence (36, 40, 41). Tumor dormancy is a critical factor in these poor outcomes. The DREAM repressor complex, whose assembly is dependent on Dyrk1A, has been implicated in cancer cell dormancy in ovarian cancer cell lines. Inhibition of Dyrk1A using compounds like Harmine and INDY blocks DREAM assembly, leading to increased DNA synthesis and cell death, and improves response to carboplatin, positioning Dyrk1A as a potential therapeutic target for epithelial ovarian cancer (EOC) treatment (27). Hypoxia is also a significant driver in OC, inducing cancer stem cell (CSC) formation and chemoresistance through the activation of the PLD2 gene by HIF-1α. PLD2 overexpression promotes stemness gene expression, correlates with reduced patient survival, and enhances resistance to cisplatin and carboplatin. Pharmacological inhibition of PLD2 restores chemosensitivity, highlighting the HIF-1α-PLD2 axis as a promising target (42). Cancer-associated fibroblasts (CAFs), particularly FAP-high subpopulations, play a crucial role in promoting drug-tolerant persister (DTP) formation in HGSOC cells upon carboplatin treatment, with ECM organization by CAFs facilitating DTP emergence, suggesting that targeting CAF-ECM interactions could overcome resistance (43). The nuclear factor-κB-inducing kinase (NIK) is elevated in ovarian cancer CSCs and is associated with high chemoresistance and relapse rates. NIK knockdown enhances sensitivity to carboplatin and paclitaxel, indicating its role in CSC maintenance and stemness, and suggesting NIK inhibition as a strategy to prevent relapse (44). Similarly, the SPC25/RIOK1/MYH9 axis is crucial for tumor stemness and platinum resistance in EOC. SPC25 forms a trimeric complex that leads to MYH9 nuclear accumulation and Wnt/β-catenin signaling activation. Inhibiting this axis with CBP1 reduces CSC phenotypes and enhances platinum efficacy, offering a potential strategy to improve platinum sensitivity and survival (45).

Immune evasion mechanisms are also linked to dormancy in OC. Egfl6 enhances the immunosuppressive functions of tumor-associated myeloid cells (MDSCs and TAMs) by binding β3 integrins and activating p38 and SYK signaling. This leads to increased intratumoral MDSCs and TAMs, upregulation of CXCL2, IL-10, and PD-L1, and can block anti-PD-L1 therapy, making EGFL6 a potential therapeutic target to enhance immunotherapy (46). STARD7 has been identified as a tumor-promoting factor in OC, activating the NF-κB signaling pathway and potentially serving as an independent prognostic indicator (47). Progesterone, a key hormone, has been associated with reduced HGSOC risk, and studies suggest it can inhibit cell migration in fallopian tube-derived models, though this effect may be lost in cells with oncogenic mutations (48). Mutant p53 aggregation, common in HGSOC, presents a novel therapeutic strategy, as p53 reactivators can decrease mutant p53 expression and shift its localization, potentially restoring normal p53 function (49). DNA topoisomerase 2-beta (TOP2B) has been identified as a potential negative regulator of the antigen presentation pathway (APP) in HGSOC, and its inhibition could lead to novel therapeutic strategies (50). Metabolic alterations in mitochondria, including changes in oxidative stress, mass, and biogenesis, are dynamic in OC, potentially challenging therapeutic targeting (51). SOX2, while crucial for dormancy in melanoma, also plays a role in promoting anchorage-independent survival of ovarian cancer cells, with its knockdown downregulating LGR5, a receptor involved in Wnt signaling and cell survival (52).

Emerging evidence highlights a bidirectional link between metabolic reprogramming in dormant ovarian cancer cells and the establishment of an immunosuppressive niche. Dormant ovarian cancer cells exhibit a distinct metabolic phenotype characterized by reduced glycolysis (53), enhanced fatty acid oxidation (FAO), and elevated NAD+ salvage pathway activity (54). This metabolic shift not only supports long-term survival under nutrient-limited conditions but also actively shapes the local immune landscape. One critical axis involves the secretion of oncometabolites and cytokines that recruit and stabilize regulatory T cells (Tregs) (53). For instance, dormant cells upregulate Dickkopf-3 (DKK3), which specifically recruits Tregs into the tumor bed (55). These Tregs, in turn, exhibit heightened oxidative phosphorylation (OXPHOS) and succinate accumulation, conferring a metabolic advantage that sustains their suppressive function even in low-glucose environments typical of dormant niches (56). The Treg-derived IL-10 and TGF-β further reinforce tumor cell quiescence by activating STAT3 and inhibiting pro-proliferative ERK signaling, creating a positive feedback loop of mutual maintenance (57). Additionally, lactate—though produced at lower levels by dormant cells—can be scavenged by neighboring Tregs and M2-like macrophages via MCT1, fueling their immunosuppressive activity. The histone lactylation-driven expression of B7-H3 on dormant cells further impairs CD8+ T cell effector function (58). Notably, this metabolic symbiosis renders dormant ovarian cancer cells resistant to immune checkpoint blockade (ICB). Preclinical studies suggest that disrupting this loop—either by inhibiting FAO (e.g., with etomoxir), blocking MCT1, or depleting Tregs—can sensitize dormant cells to ICB and chemotherapy (59).

Therefore, targeting the metabolic vulnerabilities of the dormant tumor-immune synapse represents a promising therapeutic avenue. Combinatorial strategies incorporating metabolic inhibitors (e.g., glutaminase inhibitors for NRF2-high cells, FAO inhibitors for quiescent cells) alongside Treg-depleting agents or DKK3-neutralizing antibodies may effectively eradicate the dormant reservoir and prevent late recurrence in ovarian cancer.

### 3.3 Endometrial cancer

Endometrial cancer (EC) is another common gynecologic malignancy where dormancy-related mechanisms contribute to its progression and recurrence. The ARID1A gene, frequently mutated in EC, plays a multi-faceted role in both precancer and cancer development, influencing tumor behavior and potentially dormancy, though its direct link to dormancy requires further elucidation (Morgan et al., 2024). Stromal CD10 expression has been implicated in the progression of endometrial and endometriosis-associated cancers. CD10-negative endometriosis-derived mesenchymal stem cells (enMSCs) promote clear cell carcinoma (CCC) growth by regulating iron levels, altering Ferritin L and Ferritin H balance, and increasing the labile iron pool in CCC cells, highlighting a tumor-promoting stromal cell within the endometrium that could influence dormant cell survival (60). Immune evasion in EC can be promoted by the loss of LATS1/2, which are frequently mutated and downregulated in EC. This loss is associated with significant MHC-I downregulation, independent of the Hippo-YAP pathway. LATS1/2 directly interact with and phosphorylate STAT1, enhancing MHC-I transcription. Consequently, loss of LATS1/2 confers increased resistance to immune cell-mediated killing, which can be reversed by MHC-I overexpression, suggesting LATS1/2 as a target for immune checkpoint blockade therapy in EC (61). Lymphovascular space invasion (LVSI), a critical prognostic factor in high-grade serous endometrial adenocarcinoma, involves complex interactions within the immune landscape of both the tumor and its microenvironment, with tumor cells within LVSI showing positivity for IL-12R-B2 and S100A4, indicating a role in promoting metastasis and potentially influencing dormancy within these emboli (62).

### 3.4 Uterine sarcoma

While the provided literature does not offer specific studies on tumor dormancy cells in uterine sarcoma, the general principles of dormancy observed in other gynecologic and solid tumors are likely applicable. Uterine sarcomas are aggressive malignancies with a high propensity for recurrence and metastasis, suggesting that dormant cell populations may contribute significantly to their challenging clinical course. Research into the intrinsic and extrinsic factors governing dormancy in other GCs, such as hypoxia-induced quiescence, ECM interactions, and immune evasion mechanisms, provides a framework for future investigations into uterine sarcoma. The lack of specific literature highlights a research gap, emphasizing the need for dedicated studies to understand dormancy in this particular gynecologic malignancy.

**Table 1** summarizes the core mechanisms of tumor dormancy in four gynecological cancers and their representative molecular pathways. It is evident that the regulation of dormancy exhibits significant heterogeneity across different cancer types: cervical cancer primarily relies on a hypoxia-induced reversible dormant state and radiotherapy resistance mediated by extracellular vesicles; ovarian cancer involves the most complex mechanisms, encompassing multiple pathways, such as cell cycle proteins (DREAM complex), hypoxia-related stemness (HIF-1α/PLD2), microenvironment interactions (CAF-ECM), stem cell maintenance factors (NIK, SPC25/RIOK1/MYH9), and immunosuppressive molecules (Egfl6); endometrial cancer is characterized by immune evasion due to MHC-I downregulation caused by LATS1/2 deficiency, as well as the survival of dormant cells being influenced by iron metabolism in CD10-positive stromal cells. In contrast, research on tumor dormancy in uterine sarcoma remains scarce, and the aforementioned mechanisms provide important references for future exploration.

**Table 1:** Dormancy-related mechanisms and challenges in gynecologic cancers.

Cancer type	Key dormancy-related mechanisms/Factors	Clinical implications
Cervical Cancer	Hypoxia-induced E6/E7 downregulation, mTOR impairment; apoEV-MTA1/p-STAT1/NR2F1 axis	Chemoresistance, Radioresistance, Recurrence
Ovarian Cancer	Dyrk1A/DREAM complex; HIF-1α-PLD2 axis; CAF-ECM interactions; NIK/SPC25/RIOK1/MYH9 axes	Chemoresistance, CSC formation, Recurrence
Endometrial Cancer	Stromal enMSCs/iron regulation; LATS1/2 loss/MHC-I downregulation	Tumor progression, Immune evasion
Uterine Sarcoma	(Limited specific data)	High recurrence, Metastasis

## 4 Dormancy and tumor recurrence and drug resistance

Tumor recurrence remains a formidable challenge in cancer treatment, with dormant tumor cells being a primary driver of post-treatment relapse, often occurring years after initial therapy (2–4, 29, 63). These quiescent cells evade conventional therapies that target rapidly proliferating cells, persisting as a reservoir for future disease progression.

### 4.1 Mechanisms of reactivation

The transition from dormancy to active proliferation, known as reawakening, is a complex process influenced by both intrinsic cellular changes and extrinsic microenvironmental cues. Chemotherapy itself can induce a state of dormancy, where tumor cells enter a “pseudo-senescence” to evade the cytotoxic impact, survive, and later recover self-renewal capacity, contributing to recurrence (64, 65). For instance, in breast cancer, chemotherapy-induced dormancy can be driven by the upregulation of Bcl-xL, a central survival factor in these dormant cells. Targeting Bcl-xL with inhibitors like A-1331852, especially when combined with low-dose immunogenic chemotherapy, has shown promise in preventing tumor relapse in mouse models, suggesting a strategy to eliminate disseminated dormant cells and prevent metastatic relapse (66, 67). Specific molecular pathways are critical for reawakening. NRF2 activation, often linked to oxidative stress, promotes the recurrence of dormant tumor cells by inducing metabolic reprogramming that supports redox homeostasis and nucleotide synthesis. Suppressing NRF2 can hinder recurrence, while glutaminase inhibition can target NRF2-high dormant and recurrent tumors, preventing reactivation (17). Conversely, the F-box protein Fbxw7 is crucial for maintaining DTC dormancy; its ablation can disrupt quiescence, making cells proliferative and more sensitive to chemotherapy, thereby reducing DTCs and prolonging survival in mice (13). The IGF1R signaling pathway also plays a role, as G2.2, a heparan sulfate hexasaccharide mimetic, has been shown to inhibit chemotherapy-induced dormancy and tumor recurrence in xenografts, potentially by targeting DTCs through IGF1R inhibition (31).

The tumor microenvironment (TME) significantly influences the reawakening of dormant cells. Inflammation, for example, can convert dormant cancer cells into aggressive metastases. Neutrophil extracellular traps (NETs), produced during inflammation, are required for this conversion, as their proteases cleave laminin, triggering dormant cell proliferation. Antibodies against NET-remodeled laminin can prevent this awakening, highlighting a potential therapeutic intervention (23). The aging lung microenvironment has also been shown to promote metastatic melanoma dormancy reactivation, involving Wnt5A and AXL activation and a decrease in NK cell infiltration and function (68). The bone microenvironment can also drive dormancy escape, with osteoclast-mediated bone resorption releasing growth factors and bone marrow adipocytes providing metabolic support, while immunosuppressive myeloid cells and regulatory T cells promote reawakening (24). The extracellular matrix (ECM) is a critical component of the TME that provides structural support and biochemical cues, profoundly influencing tumor cell dormancy. The composition and stiffness of the ECM can directly regulate cell adhesion, signaling pathways, and cell cycle progression. For instance, fibronectin, a key ECM protein, has been shown to maintain a dormant breast cancer population. Dormancy-inducing cells form a fibronectin matrix via integrin adhesion, ROCK tension, and TGFβ2, while cancer cell outgrowth post-dormancy requires MMP-2-mediated fibronectin degradation (21). This highlights the ECM’s role in both establishing and breaking dormancy. Beyond specific proteins, the physical properties of the ECM, such as stiffness, can also dictate cell fate. Stiffer matrices can induce dormancy and drug resistance in cancer stem cells, whereas softer matrices promote stem cell states, suggesting that mechanical forces are critical regulators in the TME (69). The ECM of targeted organs can actively maintain tumor cell dormancy or trigger reactivation, underscoring the importance of organ-specific niches in metastatic progression (22). For example, a fibrotic milieu enriched with type I collagen can induce the switch from dormancy to metastatic growth, as demonstrated in modified 3D *in vitro* systems (70). Furthermore, neutrophil extracellular traps (NETs), released during inflammation, can remodel the ECM by cleaving laminin, thereby awakening dormant cancer cells and promoting aggressive metastases (23). These findings emphasize that the ECM is not merely a passive scaffold but an active participant in regulating tumor dormancy, offering potential therapeutic targets by modulating its composition or physical properties. Inflammation, often triggered by factors like tobacco smoke or bacterial lipopolysaccharide (LPS), can awaken dormant cancer cells. Neutrophil extracellular traps (NETs), released by neutrophils during inflammation, are crucial for this conversion, as their proteases cleave laminin, triggering dormant cell proliferation (23). This mechanism suggests that targeting NETs could prevent recurrence. In gynecologic cancers, NETs also influence tumor progression by attracting tumor cells, stimulating further NET release, and increasing invasiveness and metastasis through immune modulation and epithelial-mesenchymal transition (39). Myeloid-derived suppressor cells (MDSCs) and tumor-associated macrophages (TAMs) are key immunosuppressive cells within the TME that can foster dormancy and immune evasion. MDSCs mediate immune escape and contribute to a suppressive TME, correlating with tumor stage, metastasis, and prognosis (71). Eliminating MDSCs can inhibit tumor growth and metastasis, making them attractive therapeutic targets. TAMs, particularly those expressing CD163, also play a significant role, and their dynamics during immunotherapy can be tracked using specific nanobody-based immunotracers to predict therapy responsiveness (72). CD276 (B7-H3) on TAMs can diminish antitumor immune responses by enhancing efferocytosis, a process where macrophages engulf apoptotic cells, thereby promoting immune evasion (73). In ovarian cancer, Egfl6 enhances myeloid cell immunosuppression by promoting MDSC and TAM differentiation, upregulating immunosuppressive factors, and blocking anti-PD-L1 therapy, suggesting Egfl6 as a potential target to enhance immunotherapy (46). Autophagy, a cellular recycling process, is essential for the survival of dormant tumor cells and subsequent recurrence. Studies in breast cancer models have demonstrated that autophagy is required for dormant tumor cell survival following therapy, and its inhibition, either genetically or pharmacologically (e.g., with chloroquine), can inhibit tumor recurrence and kill dormant cells, suggesting autophagy inhibition as a strategy to prevent lethal recurrence (74–76). Cancer stem cells (CSCs) are intimately linked to dormancy and recurrence, as they possess self-renewal capabilities and can persist in a quiescent state, driving tumor growth and therapy resistance. Autophagy has a dual role in CSCs, aiding their survival and resistance but also potentially restricting their growth, making its modulation a complex but promising therapeutic avenue (30, 33, 77, 78). The initiation of tumor dormancy can also be influenced by the lymphovascular embolus. In experimental models, lymphovascular emboli formation and spheroidogenesis were associated with decreased proliferation, G0/G1 cell cycle arrest, and reduced mTOR signaling. This dormancy induction required calpain-mediated E-cadherin proteolysis and decreased PI3K signaling, suggesting that these pathways are critical for initiating dormancy within emboli and preventing immediate metastatic outgrowth (79, 80). Understanding these diverse mechanisms of dormancy and reawakening is crucial for developing targeted interventions to prevent tumor recurrence in gynecologic cancers.

### 4.2 Dormancy and tumor drug resistance

One of the most profound clinical implications of tumor dormancy is its direct contribution to drug resistance, a major impediment to successful cancer treatment and a primary cause of recurrence (4, 64). Dormant cells are inherently resistant to conventional chemotherapies and radiotherapies, which primarily target rapidly proliferating cells by interfering with DNA replication or cell division (29). The quiescent nature of dormant cells is a fundamental mechanism of their drug resistance. By existing in a state of cell cycle arrest, typically G0/G1, dormant cells are largely unaffected by cytotoxic agents designed to kill dividing cells (11, 12). This reduced metabolic activity also contributes to resistance, as many drugs rely on active cellular processes for uptake or efficacy (19). Specific molecular pathways within dormant cells confer resistance. For instance, slow-cycling/dormant cancer cells (SCCs) exhibit high survival capacity and drug resistance due to the upregulation of RGS2, which causes translational arrest via eIF2α phosphorylation. RGS2 antagonism or the use of phosphodiesterase 5 inhibitors like sildenafil can induce apoptosis in SCCs under stressed conditions, suggesting a strategy to sensitize dormant cells to chemotherapy (15). NRF2 activation, which promotes redox homeostasis and nucleotide synthesis, also contributes to drug resistance in dormant cells, making glutaminase inhibition a potential strategy to target NRF2-high resistant tumors (17). Bcl-xL has been identified as a central survival factor in chemotherapy-induced dormancy, and its molecular targeting can inhibit breast cancer relapse, highlighting its role in drug resistance (66, 67). Furthermore, a dormancy-inducing 3D engineered matrix has uncovered a mechanosensitive and drug-protective FHL2-p21 signaling axis, where FHL2 nuclear localization leads to high p21Cip1/Waf1 expression, promoting growth arrest and chemotherapy resistance. Downregulating FHL2 sensitizes cells to chemotherapy, suggesting its role in resistance (81).

The tumor microenvironment (TME) plays a crucial role in fostering drug resistance in dormant cells. Hypoxia, a common feature of the TME, leads to dormant tumor cells that are resistant to chemotherapy, as seen in HPV-positive cancers where it induces a reversible growth arrest that protects cells against therapies (11, 20). Cancer-associated fibroblasts (CAFs) can promote the emergence of drug-tolerant persister (DTP) cells in ovarian cancer, with the extracellular matrix (ECM) deposited by CAFs facilitating this resistance (43). The ECM, through its interactions with hyaluronan (HA), can modulate chemoresistance by activating signaling pathways and influencing tumor stroma properties, contributing to epithelial-mesenchymal transition (EMT) and resistance. Targeting HA synthesis, degradation, or CD44 binding could counteract chemoresistance (82). Matrix stiffness, a physical characteristic of the TME, can induce dormancy and resistance in cancer stem cells, while softer matrices promote stem cell states, revealing the importance of mechanical forces in the TME for drug resistance (69).

Metabolic alterations are also key to drug resistance. Dysregulated sphingolipid metabolism, for instance, promotes cancer cell survival by enhancing drug efflux, with upregulated glucosylceramide synthase (GCS) and ABCB1 gene expression contributing to this resistance. Targeting GCS, sphingosine kinase, and acid ceramidase could enhance chemotherapeutic efficacy (83). NAD+ metabolism is crucial for metabolic and signaling pathways in gynecologic cancers, regulating growth and metabolism. NAD+ synthesis enzymes are key targets for therapy, as their dysregulation can contribute to resistance (84).

Epigenetic modifications, including DNA methylation, histone modification, and non-coding RNAs, contribute significantly to cisplatin resistance. These epigenetic alterations can predict response to platinum agents and offer targets for epigenetic therapies to enhance cancer cell susceptibility to drugs (85). Cancer stem cells (CSCs) are central to therapy resistance due to their inherent plasticity, self-renewal capacity, and ability to enter dormancy. Autophagy, for example, has a dual role in CSCs, aiding their survival and resistance to chemotherapy and radiotherapy. Modulating autophagy with inhibitors or enhancers shows potential in making CSCs more responsive to standard treatments (33, 75–78). Patient-derived tumor organoids (PDOs), combined with single-cell analysis, lineage tracing, and functional assays, offer a unique system to investigate cancer cell plasticity, including drug-tolerant persister cells and CSC states, which are crucial for overcoming therapeutic resistance (34). The fusion of tumor cells and mesenchymal stem/stroma cells can also contribute to tumor heterogeneity, cancer stem cells, and therapy resistance, highlighting the role of cell fusion in tumor survival and evolution (86).

Therapy-induced senescence (TIS) is another complex factor. While TIS can lead to resistance and recurrence, it can also enhance therapy response and anti-tumor immunity. Senescent cells (SnCs) express the senescence-associated secretory phenotype (SASP), which modulates the TME and mediates both resistance and response to therapies. Strategies to mitigate senescence, such as modulating SASP or targeting SnC persistence, may improve cancer treatment benefits (87). Overall, the multifaceted nature of drug resistance in dormant cells necessitates innovative therapeutic strategies that either eliminate these cells, induce permanent dormancy, or reawaken them to resensitize them to conventional treatments.

In the context of gynecologic tumors, specific mechanisms linking dormancy to recurrence and chemoresistance are emerging. As mentioned, in cervical cancer, MTA1 in apoptotic tumor cell-derived extracellular vesicles (apo EVs) mediates radio resistance by inducing cellular dormancy through the p-STAT1/NR2F1 signaling axis (37). This suggests that targeting this pathway could enhance radiosensitivity. In ovarian cancer, the tumor microenvironment (TME) significantly influences chemoresistance, with metabolic reprogramming playing a key role (40). Cancer-associated fibroblasts (CAFs), for example, can promote the emergence of drug-tolerant persister (DTP) cells in high-grade serous ovarian cancer (HGSC) upon carboplatin treatment, with ECM organization by FAP-high CAFs facilitating DTP formation (43). These DTPs often exhibit dormancy-like characteristics, contributing to relapse.

The intrinsic molecular pathways that govern dormancy, such as the ERK/p38 balance, mTOR signaling, and the activity of transcription factors like NR2F1, are also implicated in drug resistance. NR2F1, a dormancy-associated transcription factor, is a key determinant of therapeutic resistance, and its overexpression can reduce therapeutic efficacy while sustaining mTORC1 transcriptional regulation (16). While this study was in melanoma, the principles are highly relevant to gynecologic cancers, where similar pathways may operate. The reversible nature of dormancy, influenced by treatment dose and patient differences, underscores the need for strategies that either eliminate dormant cells or induce permanent dormancy to achieve better outcomes (64). Emerging therapeutic strategies aim to target dormant cells directly, maintain their quiescent state, or re-awaken them to sensitize them to conventional therapies (7, 63, 88). For example, G2.2, a heparan sulfate hexasaccharide mimetic, has shown promise in inhibiting chemotherapy-induced dormancy and preventing tumor recurrence in xenograft models, potentially by targeting DTCs through IGF1R inhibition (31). Similarly, targeting Bcl-xL, a survival factor upregulated in dormant tumor cells, with inhibitors like A-1331852, combined with chemotherapy, can inhibit tumor relapse (66, 67). These findings highlight the critical link between dormancy and therapeutic failure in gynecologic cancers and emphasize the need for dormancy-specific interventions.

## 5 Dormancy and immune evasion

Tumor dormancy cells pose a significant challenge to effective cancer treatment not only due to their resistance to conventional therapies but also their remarkable ability to evade immune surveillance, contributing to disease persistence and recurrence (89, 90). This immune evasion is a complex process involving both intrinsic cellular mechanisms and the creation of an immunosuppressive tumor microenvironment (TME).

### 5.1 Mechanisms of immune evasion by dormant cells

Dormant disseminated tumor cells (DTCs) can evade endogenous immunity primarily due to their scarcity and altered antigen presentation (89). One key mechanism involves the indoleamine 2,3-dioxygenase 1 (IDO1)-kynurenine-AhR-p27 pathway, which is induced by IFN-γ and leads to tumor-repopulating cell (TRC) dormancy. This pathway prevents STAT1 signaling, activating dormancy and allowing immune evasion. Blocking the IDO/AhR circuitry abrogates IFN-γ-induced dormancy and enhances tumor regression, suggesting that combining IFN-γ with IDO1 inhibitors could be a potential cancer immunotherapy (90). Altered antigen presentation is a common strategy for immune evasion. In endometrial cancer, loss of LATS1/2, frequently mutated and downregulated, promotes immune evasion by significantly downregulating MHC-I expression, independent of the Hippo-YAP pathway. LATS1/2 directly interact with and phosphorylate STAT1, enhancing MHC-I transcription. Consequently, LATS1/2 loss confers increased resistance to immune cell-mediated killing, which can be reversed by MHC-I overexpression, suggesting LATS1/2 as a target to enhance immune checkpoint blockade therapy (61). Similarly, NPM1 inhibits tumoral antigen presentation by associating with IRF1, sequestering it from Nlrc5 and Ciita promoters, thereby suppressing MHC-I and MHC-II expression. High NPM1 expression correlates with low survival rates, and NPM1 deficiency inhibits tumor progression and enhances survival, indicating NPM1 as a potential target for cancer immunotherapy (91). DNA topoisomerase 2-beta (TOP2B) has also been identified as a potential negative regulator of the antigen presentation pathway (APP) in high-grade serous ovarian cancer (HGSOC), and its inhibition could lead to novel therapeutic strategies (50).

### 5.2 Immunosuppressive tumor microenvironment

Dormant cells actively shape an immunosuppressive TME that further facilitates immune evasion. Myeloid-derived suppressor cells (MDSCs) and tumor-associated macrophages (TAMs) are key players in this process. MDSCs mediate immune escape by immunosuppression and contribute to a suppressive TME, with their levels correlating with tumor stage, metastasis, and prognosis. Eliminating MDSCs inhibits tumor growth and metastasis, making them therapeutic targets for immunotherapy (71). In ovarian cancer, Egfl6 enhances the immunosuppressive functions of tumor-associated myeloid cells (MDSCs and TAMs) by promoting their differentiation and upregulating immunosuppressive factors like CXCL2, IL-10, and PD-L1. Egfl6 can block anti-PD-L1 therapy, and its neutralization restores efficacy, highlighting it as a potential therapeutic target (46). TAMs, particularly CD163-expressing macrophages, can be tracked using specific immunotracers, offering a tool to study their dynamics during immunotherapy and predict treatment responsiveness (72). CD276 on TAMs diminishes antitumor immune response by blocking efferocytosis and enhancing MHCII expression, increasing T cell infiltration. CD276 activates lysosomal signaling and JUN to regulate AXL and MerTK, enhancing efferocytosis. Blocking CD276 and PD-1 synergistically restrains tumor growth, suggesting TAMs’ CD276 expression promotes immune evasion (73). Regulatory T cells (Tregs) also contribute to immunosuppression. In aged epithelial ovarian cancer mice, increased Treg cells exhibited enhanced immunosuppression, higher IL10 and TGFβ expression, and suppressed CD4 and CD8 T cells, linked to increased OXPHOS and succinate levels. Targeting the Treg-succinate-FGF21 pathway may be actionable in elderly EOC patients (92). Neutrophils, recruited to tumors as tumor-associated neutrophils (TANs), exhibit dual roles, influencing tumor growth, metastasis, and angiogenesis. TANs interact with the TME and undergo phenotype transitions, impacting immune evasion. Strategies targeting TANs, such as inhibiting their tumor-promoting effects or reprogramming them, are being explored for immunotherapy (93). As mentioned earlier, neutrophil extracellular traps (NETs) can awaken dormant cancer cells and also influence gynecologic cancer progression by modulating tumor immune responses and triggering epithelial-mesenchymal transition (23, 39). Immune checkpoints are critical regulators of immune evasion. CD96 blockade in anti-EpCAM CAR-T cells has been shown to eliminate dormant tumor cells in colorectal cancer, enhance CAR-T cell memory formation, and inhibit T cell exhaustion, suggesting targeting CD96 as a checkpoint to prevent disease relapse (94). VISTA, a negative immune checkpoint, inhibits T-cell activation in cancer, and high VISTA levels in immune cells hinder tumor response, promoting cancer growth. Targeting VISTA may enhance the immune system’s cancer-killing ability (95). B7-H3 (CD276), particularly its 4Ig isoform, is expressed in gynecological cancers and mediates enhanced proliferation and tumorigenic signaling through dimerization, contributing to immune evasion (96). Lysine lactylation (Kla) promotes B7-H3 expression in immune-evading tumors, with lactate treatment inhibiting CD8+ T cell antitumor immunity. Lactate-induced H3K18la binds to the B7-H3 promoter, increasing B7-H3 expression and tumor progression, suggesting glycolysis and B7-H3 inhibition can enhance anti-PD-1 efficacy (58). The cGAS/STING pathway, while involved in antitumor immunity, also has a complex role in dormancy and can be activated in early invasive disease in ovarian cancer, potentially driving progression (97, 98). Metabolic reprogramming within the TME is a significant contributor to immune evasion. Tumor cells alter the TME to modulate immune cell functions, creating an immunosuppressive environment by inhibiting effector T-cells and expanding regulatory T-cells and MDSCs. Metabolic alterations lead to cytokine and chemokine imbalance, enhancing immunosuppression (99). Lactic acid, a byproduct of tumor metabolism, suppresses immune cells via pH drop, modulates immune cell surface molecules, and enhances immunosuppressive cells. Inhibiting lactic acid production or blocking transporters, combined with immunotherapies, could improve tumor immune evasion (100). Metabolic shifts in immune cells like T cells, macrophages, dendritic cells, and MDSCs, affecting glucose, lipid, and amino acid metabolism, are crucial for developing novel cancer therapeutic strategies to enhance anti-tumor activities and prevent drug resistance (101).

There is an active, immunological symbiotic relationship between tumor dormant cells and regulatory T cells (Tregs), which constitutes a key mechanism for tumor immune escape and long-term dormancy maintenance. Specifically, dormant tumor cells specifically recruit Treg cells into their microenvironment through high expression and secretion of the Dickkopf-3 (DKK3) protein, forming a Treg-dominated immune immune barrier (55). These recruited Treg cells secrete inhibitory cytokines such as IL-10 and TGF-β and competitively consume IL-2, effectively inhibiting the activation and proliferation of CD8^+^ cytotoxic T lymphocytes, thereby protecting dormant tumor cells from immune clearance (102). This mechanism not only allows tumor cells to lie dormant in the host body for a long time, but also lays hidden dangers for future recurrence and metastasis. Further studies have shown that intervention on this signaling axis—such as knockdown of DKK3 expression or specific clearance of tumor local Treg cells—can significantly de-immunosuppress and restore the killing function of CD8^+^ T cells, thereby clearing more than 60% of dormant tumor cells (102, 103). Therefore, targeting DKK3-Treg, an immune regulatory pathway, is expected to become an important new strategy to prevent tumor recurrence after dormancy and improve the efficacy of existing immunotherapies. Epigenetic aberrations in cancer cells reprogram the TME, hindering antitumor immunity and promoting tumor progression. Targeting epigenetically mediated tumor-immune crosstalk is a strategy to inhibit tumor progression and overcome immunotherapy limitations (104). DNA methylation, in particular, influences immune cell function and tumor immune evasion, regulating immune cell differentiation and responses. Modifying DNA methylation can enhance immune cell infiltration and function, advancing tumor immunotherapy (105). Aberrant R-loop-mediated immune evasion, cellular communication, and metabolic reprogramming also affect cancer progression. Low R-loop scores in malignant cells activate glycolysis, EMT, and immune escape, suggesting R-loop regulators as potential targets for precision medicine (106).

Extracellular vesicles (EVs) also play a role in immune modulation and tumor aggressiveness. Exosomal noncoding RNAs (ncRNAs) are linked to tumor progression and drug resistance, modulating tumorigenesis, metastasis, and the TME, and have potential as diagnostic and prognostic biomarkers (107). EVs from aggressive ovarian cancer cells contain UBE2NL and HIST2H3PS2, which promote tumor aggressiveness and metastasis in gynecologic cancers, suggesting them as prognostic biomarkers and therapeutic targets (108).

Finally, the scarcity of dormant cells makes them difficult targets for immune surveillance, yet T cell immunotherapies can overcome this by targeting MHC-restricted and -unrestricted DTC antigens (89). Integrated biomarker profiling, including TGF-β and CD47, and metabolic models, such as pyruvate transport and folate metabolism, can predict immunotherapy outcomes in gynecologic cancer patients, aiding clinical decision-making and personalized treatment (109). Single-cell transcriptome analysis in colorectal cancer has revealed T cell subsets associated with therapeutic resistance, linked to altered antigen processing and presentation pathways, and transcriptional network dysregulation, offering insights applicable to gynecologic cancers (110). MicroRNAs (miRNAs) also regulate gene expression in cancer, influencing tumor growth and immune response, with some enhancing immune attack and others aiding cancer cell immune evasion. miRNAs may predict immunotherapy response and serve as novel treatment components (111).

**Figure 1:**
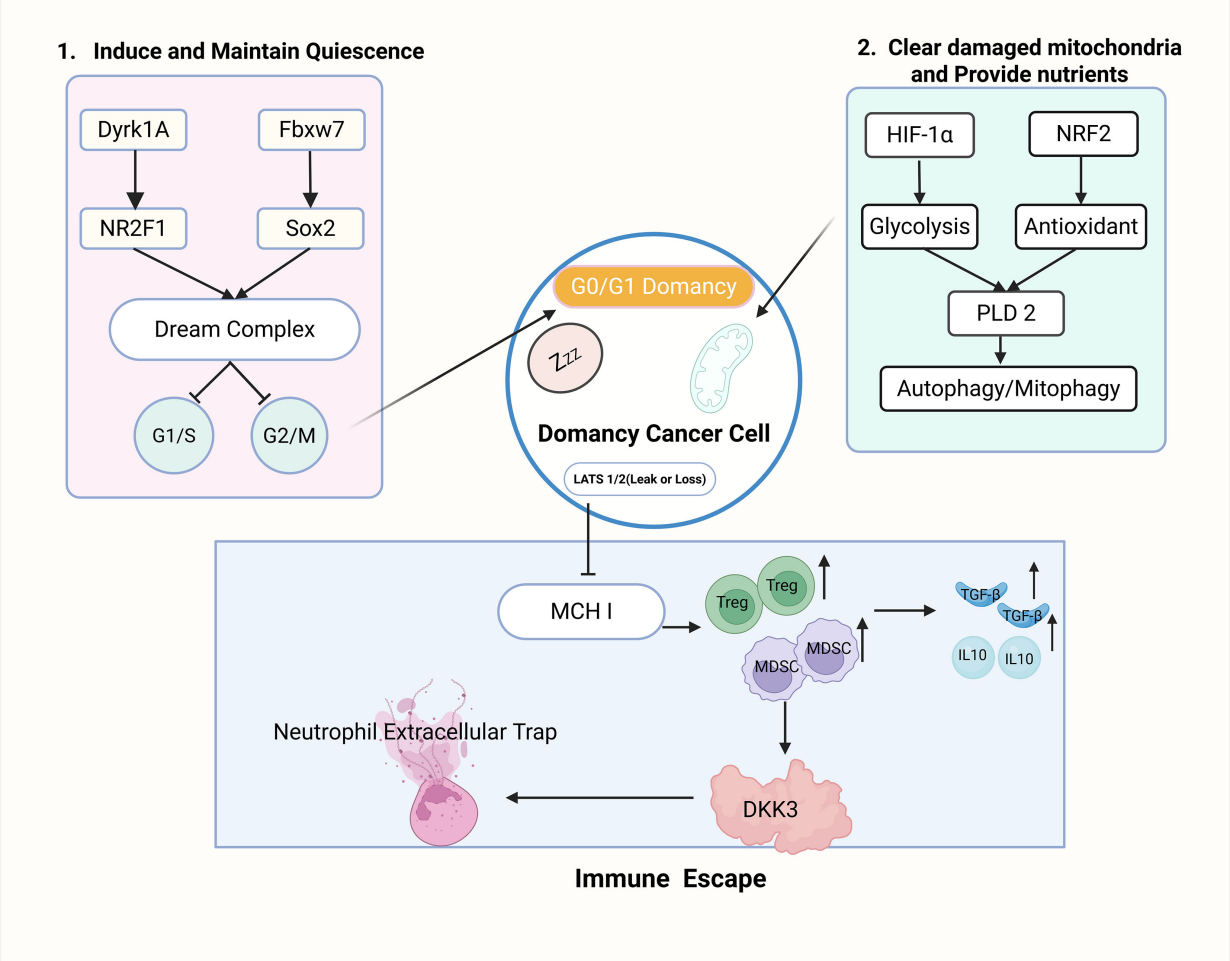
Mechanisms of tumor dormancy and drug resistance.

As summarized in **Figure 1**, the complex landscape of tumor dormancy can be conceptually unified into three interconnected regulatory modules: intrinsic quiescence maintenance, extrinsic microenvironmental sensing, and immune-metabolic adaptation. Intrinsic Module: Key regulators such as DREAM complex (Dyrk1A), enforce quiescence. Extrinsic Module: Microenvironmental cues HIF-1α, NRF2 regulate dormancy entry. Immune evasion and therapy resistance Module: Mechanisms like MHC-I downregulation (via LATS1/2 loss) confer immune escape and drug tolerance.

## 6 Therapeutic strategies and future directions

The profound implications of tumor dormancy for recurrence, drug resistance, and immune evasion in gynecologic cancers necessitate the development of innovative therapeutic strategies. Current research focuses on several key approaches: directly eliminating dormant cells, inducing permanent dormancy, reawakening dormant cells to sensitize them to conventional therapies, and enhancing immune responses against these elusive populations (3, 4, 7, 63).

### 6.1 Targeting dormant cells

Direct elimination of dormant cells is a highly desirable but challenging goal. Strategies include targeting specific survival pathways or vulnerabilities unique to the dormant state. For instance, G2.2, a synthetic mimetic of heparan sulfate hexasaccharide, has shown promise in inhibiting chemotherapy-induced dormancy and preventing tumor recurrence by targeting DTCs, potentially through IGF1R inhibition (31). Given that Bcl-xL is a central survival factor in chemotherapy-induced dormancy, its inhibitors, such as A-1331852, are being investigated to eradicate disseminated dormant cells and prevent metastatic relapse, particularly when combined with immunogenic chemotherapy (66, 67). Blockade of CD96, an immune checkpoint, has been shown to eradicate dormant tumor cell recurrence in a T cell-dependent manner, suggesting a novel immunotherapeutic approach to eliminate dormant cells (94). In ovarian cancer, inhibition of Dyrk1A, which is essential for DREAM complex assembly, leads to increased DNA synthesis and cell death, improving carboplatin response and offering a therapeutic target for EOC treatment (27). Glutaminase inhibition can target NRF2-high dormant and recurrent tumors, preventing reactivation by disrupting metabolic reprogramming (17). Furthermore, RGS2 antagonism or phosphodiesterase 5 inhibitors like sildenafil can induce apoptosis in slow-cycling/dormant cancer cells under stressed conditions, making them susceptible to low-dose chemotherapy (15). Another strategy involves inducing permanent dormancy, thereby preventing reactivation and subsequent recurrence. This approach aims to maintain cells in a quiescent, non-proliferative state indefinitely, effectively converting a lethal disease into a manageable chronic condition (7, 9). Understanding the precise molecular switches that maintain dormancy, such as the balance between ERK/p38 signaling, epigenetic remodeling, and metabolic adaptations, is crucial for developing such interventions (3).

Alternatively, reawakening dormant cells and sensitizing them to conventional chemotherapy is a viable strategy. By forcing quiescent cells back into the cell cycle, they become vulnerable to cytotoxic agents. Ablating Fbxw7 in breast cancer cells, for example, disrupts DTC quiescence, making them proliferative and more sensitive to chemotherapy like paclitaxel, thereby reducing DTCs and prolonging survival (13). Translation-instigating pharmacological interventions, when combined with low-dose chemotherapy, have shown effectiveness in preventing tumor progression in NSCLC patients by targeting dormant cells (15). Autophagy, being essential for dormant tumor cell survival and recurrence, can be targeted by inhibitors like chloroquine, which has been shown to inhibit tumor recurrence and kill dormant cells, suggesting autophagy inhibition as a strategy to prevent lethal recurrence (74–76).

### 6.2 Overcoming drug resistance and enhancing immunotherapy

Beyond direct targeting of dormant cells, overcoming drug resistance and enhancing immunotherapy are critical for improving outcomes in gynecologic cancers. Combination therapies that address the multifaceted nature of resistance are essential. This includes targeting the tumor microenvironment (TME), cancer stem cells (CSCs), epigenetic modifiers, and metabolic pathways. For instance, targeting the HIF-1α-PLD2 axis in ovarian cancer can restore sensitivity to cisplatin and carboplatin (42). Overcoming CSC-driven resistance requires advanced strategies, including combination therapies and immunotherapies, with artificial intelligence aiding in designing personalized CSC-targeted therapies (33, 34, 77, 78). Modulating autophagy in CSCs with inhibitors or enhancers can make them more responsive to standard treatments (75, 77). Epigenetic targeting therapies can enhance cancer cell susceptibility to platinum drugs by reversing epigenetic modifications that contribute to resistance (85). Targeting the SPC25/RIOK1/MYH9 axis in EOC can improve platinum sensitivity and survival (45).

Enhancing immunotherapy against dormant and resistant cells is a promising avenue. This involves targeting immune checkpoints, MDSCs, TAMs, antigen presentation pathways, and metabolic vulnerabilities. Blockade of CD96, as mentioned, can prevent disease relapse by eliminating dormant tumor cells (94). Combining IFN-γ with IDO1 inhibitors shows potential in cancer immunotherapy by abrogating IFN-γ-induced dormancy (90). Targeting MDSCs and TAMs, which mediate immune escape, can improve treatment response and survival (46, 71, 73). Reprogramming CAFs and TAMs, altering tumor metabolism, and addressing genomic alterations are also crucial for overcoming immunotherapy resistance (112). Targeting VISTA, a negative immune checkpoint, may enhance the immune system’s cancer-killing ability (95). Inhibiting glycolysis and B7-H3 can suppress tumor growth and enhance anti-PD-1 efficacy by reducing lactate-induced H3K18la and B7-H3 expression (58). The use of HPK1 inhibitor FB849 has shown promise in reinvigorating exhausted tumor-infiltrating CD8 T cells and synergizing with anti-PD-1 blockade in gynecologic malignancies, particularly endometrial cancer, supporting clinical trials (113). MicroRNAs (miRNAs) may predict immunotherapy response and serve as novel treatment components by influencing immune cell behavior and inflammation (111).

### 6.3 Novel technologies and biomarkers

Advanced technologies are revolutionizing the study and targeting of dormancy. Organoid technology provides patient-derived models for understanding disease mechanisms, drug discovery, and personalized therapeutic strategies in gynecologic cancers (1, 34). Nanoparticles are being engineered for gynecologic cancer therapy to overcome barriers like systemic toxicity and stromal fibrosis, enabling targeted drug delivery, nucleic acid delivery, and immunotherapy to restore antitumor immune function (36). Bioprinting allows for the creation of high-throughput 3D co-culture models to screen therapeutics and identify dormancy regulation pathways (35). Single-cell analysis, combined with AI, is providing a comprehensive understanding of cancer dormancy, including identifying regulatory programs associated with tumor resistance during immunotherapy. The identification of reliable biomarkers for dormancy and recurrence is crucial for clinical translation. Circulating tumor DNA (ctDNA) holds potential for use in ovarian, endometrial, and cervical cancers, though more research is needed for routine adoption (114). Long non-coding RNA (lncRNA) H19 contributes to gynecologic cancers, acting as both an oncogene and tumor suppressor depending on the cancer type, and its expression levels correlate with clinical parameters and patient outcomes, making it a potential biomarker (115). Extracellular vesicles (EVs) and their associated ncRNAs also have potential as diagnostic and prognostic biomarkers for gynecologic tumor drug resistance (107). Specific genes identified in dormant cells, such as EFNB2, PTTG1IP, and TNFRSF11A, may aid in lung adenocarcinoma diagnosis and could be explored in gynecologic contexts (32).

## 7 Discussion

While the field of tumor dormancy has made substantial progress in elucidating its fundamental mechanisms, several critical limitations persist, particularly concerning its specific role in gynecologic cancers. These gaps highlight the urgent need for further investigation and provide clear entry points for the current study. Firstly, a significant portion of the foundational research on tumor dormancy has been conducted in breast cancer, prostate cancer, and melanoma models. While many molecular and cellular mechanisms of dormancy are likely conserved across cancer types, the unique biological characteristics, microenvironments, and metastatic patterns of gynecologic tumors may lead to distinct dormancy programs. For instance, ovarian cancer’s peritoneal dissemination and the specific immune landscape of the pelvic cavity present unique challenges and opportunities for dormancy regulation that are not fully captured by models of bone or lung metastasis. The existing literature provides a general understanding of dormancy, but specific, in-depth studies focusing on cervical, ovarian, endometrial cancers, and uterine sarcomas are comparatively limited. The provided literature, while comprehensive on dormancy, only sparsely connects specific dormancy mechanisms to gynecologic cancers, with a few exceptions like cervical cancer radio resistance or ovarian cancer chemoresistance via CAFs. Secondly, while the interplay between the tumor microenvironment (TME) and dormancy is well-established, the precise components and dynamics within the gynecologic TME that regulate dormancy are not fully characterized. Studies have highlighted the importance of ECM, hypoxia, and immune cells in general dormancy (Albrengues et al., 2018; Barney et al., 2020; Butturini et al., 2019; Xiao et al., 2026). However, the specific cellular and molecular constituents of the ovarian, cervical, or endometrial TME that induce or maintain dormancy, or trigger reactivation, require more detailed investigation. For example, while the role of CAFs in ovarian cancer chemoresistance is noted (Ng et al., 2024), their specific contribution to inducing or maintaining dormancy in ovarian cancer cells needs further exploration. Similarly, the unique immune cell populations and their metabolic reprogramming within the gynecologic TME, and how they interact with dormant tumor cells to promote immune evasion, are areas ripe for deeper analysis. Thirdly, the clinical translation of dormancy-targeted therapies remains a significant hurdle. Despite promising preclinical findings for various dormancy-targeting agents and immunotherapies, their application in gynecologic oncology is still in its nascent stages. A major limitation is the lack of reliable biomarkers to identify dormant cells in patients and to monitor their response to dormancy-specific interventions. Without such markers, it is challenging to select appropriate patients for dormancy-targeted therapies or to assess their efficacy. The development of advanced methodologies, such as single-cell analysis and liquid biopsies, offers potential avenues for biomarker discovery, but their integration into routine clinical practice for dormancy detection in gynecologic cancers is yet to be realized. Finally, the mechanisms of immune evasion by dormant cells, while generally understood, need to be specifically elucidated within gynecologic cancers. The literature highlights various immune evasion strategies, including the role of MDSCs, TAMs, immune checkpoints, and metabolic reprogramming of immune cells. However, how these mechanisms are uniquely exploited by dormant cells in the context of cervical, ovarian, or endometrial cancer, and how they contribute to the limited efficacy of current immunotherapies in these diseases, is not fully clear. For instance, while HPK1 inhibitors show promise in reinvigorating exhausted T cells in endometrial cancer, their impact on dormant cell populations and their immune evasion strategies need further investigation. In summary, while the broad landscape of tumor dormancy is increasingly understood, its specific nuances within gynecologic tumors, particularly concerning the unique TME, precise molecular drivers of recurrence and drug resistance, and tailored immune evasion strategies, remain largely underexplored. This research aims to address these critical gaps by focusing on the role of tumor dormancy cells in gynecologic tumors, thereby laying a foundation for the development of more effective diagnostic tools and therapeutic interventions for these challenging malignancies.

## 8 Conclusion

Tumor dormancy cells represent a formidable biological barrier in the effective management of gynecologic cancers, fundamentally contributing to the persistent challenges of disease recurrence, therapeutic resistance, and immune evasion. This review has systematically elaborated on the intricate mechanisms governing dormancy, highlighting its critical role across cervical, ovarian, and endometrial cancers. We have seen that dormancy is not a passive state but an active, dynamic process orchestrated by a complex interplay of intrinsic cellular programs, including cell cycle arrest, specific gene regulatory pathways (e.g., Fbxw7, Sox2, RGS2, NR2F1, NRF2, MacroH2A), epigenetic modifications, and metabolic adaptations like mitophagy. Simultaneously, extrinsic cues from the tumor microenvironment, such as ECM interactions, hypoxia, inflammation, and the presence of various immune and stromal cells, profoundly influence the initiation, maintenance, and reawakening of dormant cells.

In specific gynecologic malignancies, dormancy manifests with unique characteristics. In cervical cancer, hypoxia-induced dormancy in HPV-positive cells and MTA1-mediated radioresistance through the p-STAT1/NR2F1 axis underscore the need for targeted interventions. Ovarian cancer, notorious for its high recurrence rates, sees dormancy driven by factors like the DREAM complex, hypoxia-induced PLD2, CAF-mediated drug tolerance, and the NIK/SPC25/RIOK1/MYH9 axes, all contributing to profound chemoresistance. Endometrial cancer exhibits immune evasion through LATS1/2 loss and the complex immune landscape within lymphovascular emboli. While specific research on uterine sarcoma dormancy is limited in the provided literature, the overarching principles of dormancy undoubtedly apply, necessitating dedicated investigation into this aggressive malignancy.

The contribution of dormant cells to tumor recurrence is undeniable, with chemotherapy-induced dormancy often preceding relapse. Reactivation is driven by pathways like NRF2 and influenced by microenvironmental factors such as NETs and the aging niche. Autophagy emerges as a critical survival mechanism for dormant cells, making its inhibition a promising strategy to prevent recurrence. Furthermore, the inherent drug resistance of dormant cells stems from their quiescent state, reduced metabolic activity, and specific molecular pathways (e.g., RGS2, Bcl-xL, FHL2-p21). The immunosuppressive TME, shaped by MDSCs, TAMs, Tregs, and NETs, alongside altered antigen presentation (e.g., IDO-kynurenine-AhR, MHC-I/II downregulation, NPM1) and immune checkpoint activation (e.g., CD96, VISTA, B7-H3), allows dormant cells to evade immune surveillance effectively. Metabolic reprogramming and epigenetic aberrations further contribute to this immune escape.

Despite significant advancements in understanding tumor dormancy, several research gaps remain. A major challenge lies in the lack of reliable clinical methods for detecting dormant cells in patients, which hinders early intervention and personalized treatment. The precise mechanisms governing dormancy in less studied gynecologic cancers, such as uterine sarcoma, require further elucidation. Moreover, translating promising preclinical findings into effective clinical therapies remains a hurdle, demanding robust clinical trials for dormancy-specific targets.

Future directions must focus on integrated multi-omics approaches, combining single-cell sequencing, proteomics, and epigenomics, to comprehensively map the dormant cell landscape and identify novel vulnerabilities. The development of advanced preclinical models, including patient-derived organoids and bioprinted 3D systems, will be crucial for accurately recapitulating the human TME and testing dormancy-targeting agents. Therapeutic strategies should prioritize combination approaches that simultaneously target dormant cell survival, reawaken them for conventional therapy, and enhance anti-tumor immunity. This includes developing novel small molecules, immunotherapies, and nanoparticle-based delivery systems. Ultimately, a deeper understanding of tumor dormancy and its intricate interplay with recurrence, drug resistance, and immune evasion will pave the way for innovative diagnostic tools and therapeutic interventions, offering renewed hope for improving long-term outcomes for patients with gynecologic cancers.
